# Using the Reflective Journal to Improve Practical Skills Integrating Affective and Self-Critical Aspects in Impoverished International Environments. A Pilot Test

**DOI:** 10.3390/ijerph18168876

**Published:** 2021-08-23

**Authors:** María Teresa Murillo-Llorente, Olga Navarro-Martínez, Vanessa Ibáñez-del Valle, Marcelino Pérez-Bermejo

**Affiliations:** 1 SONEV Research Group, School of Medicine and Health Sciences, Catholic University of Valencia San Vicente Mártir, C/Quevedo nº 2, 46001 Valencia, Spain; mt.murillo@ucv.es; 2 School of Medicine and Health Sciences, Catholic University of Valencia San Vicente Mártir, C/Quevedo nº 2, 46001 Valencia, Spain; olga.navarro@ucv.es (O.N.-M.); vanessa.ibanez@ucv.es (V.I.-d.V.)

**Keywords:** competency-based education, impoverished environments, international practices, nursing education, reflective journal, reflective writing

## Abstract

The reflective practice journal is a teaching methodology that facilitates the acquisition of professional, attitudinal values and skills, affording comprehensive training by reflecting on experiences that have been lived and showing feelings that, a priori, would be hidden. Our aim was to implement it in the international practicum in impoverished environments as a facilitating instrument of meaningful learning and the acquisition of professional skills, integrating affective and self-critical aspects. The project was developed with Nursing students at Catholic University of Valencia, in an impoverished environment. Qualitative reflections increased, highlighting humanity, closeness and attention focused on health promotion. The quality of the experience was 9.46/10. The mean score in self-criticism and expression was 4.57/5, and the self-evaluation of the acquisition of skills was 9.55/10. The double-blind peer evaluation of the performance of their practices in the international context was 9.68/10. The trust of the students with the teachers was evaluated as 10/10. The reflective practice journal facilitated the progression of learning, self-criticism, and the acquisition of values such as hospitality, the quality of care offered, and respect for customs and beliefs, as well as responsibility in the distribution of available resources and interventions.

## 1. Introduction

Clinical practice is fundamental in health science undergraduate studies as it complements the curriculum by developing practical skills in dealing with patients. The Bologna Declaration required universities to orient higher education towards obtaining competences [1]. Consequently, universities had to develop new methodologies and evaluating techniques, because learning from practice requires an intentional and sequential habit [2].

The aim of this work is to implement the reflective practice journal (RPJ) in the international practicum of our Nursing students in impoverished environments as an instrument of meaningful learning and the acquisition of professional skills, integrating affective and self-critical aspects.

The RPJ is a self-training assessment instrument in which students are the center of their own training process. The RPJ provides comprehensive training by reflecting on what has been experienced, showing feelings that, a priori, would be hidden. It is a tool that helps to learn and develop critical thinking and is not limited to health sciences [3]. It contributes to the development of self-confidence, self-knowledge, critical thinking, improvement in coping with conflictive situations, knowledge of deficiencies, and the care offered to patients. In addition, it also helps the teacher to know about the deficiencies of their students and allows them to implement strategies to alleviate them in real time [4].

The journal encourages reflection on practical experience, in addition to providing perspective when reading about what has been written. The work carried out by the students with respect to the description of their own experience helped them to organize their knowledge and experience. Therefore, it constitutes a necessary instrument for achieving better training of health science students since it facilitates the acquisition of professional and attitudinal competences in the individual [2,5,6]. The exercise of observing and writing about what is experienced makes the student more aware and leads to constructive criticism by detecting and analyzing situations to promote changes for improvement [7,8].

The communication of the student/teacher is bidirectional, in which the teacher accesses (preserving confidentiality) the reflections, experiences, and feelings of their students, thereby knowing in depth if they are acquiring the expected learning results, marking strategies for continuous improvement in real time. Each student has the opportunity to reflect on the development of competences in the face of real situations that arise each day in practice, offering their personal point of view, their observations, feelings, interpretations, hypotheses, and explanations that can lead them to consider changes in their actions to modify certain risky or inappropriate situations in the course of their profession [9].

To date, many studies have focused on the use of reflective practice in nursing education, as can be read in the review by Dubé and Ducharme [10]. However, only a few have examined the application of the reflective journal in the international practicum setting [11,12]. While the RPJ has been often used in the classroom, or in controlled practical experiences (e.g., in hospitals, care centers, etc.), in this work, we use RPJ in a fairly different context: the students applied the RPJ in a volunteer foreign experience in an internship health campaign in Peru.

## 2. Materials and Methods

### 2.1. Study Sample

The project was developed with students in the last year of their Nursing degree at the Catholic University of Valencia, Spain, selected from among those who voluntarily agreed to carry out their internships in an impoverished environment, specifically Requena, Peru. Seven final-year students of the Nursing degree course took the International Practicum, after a selection process was carried out among 17 candidates. Four women (57.1%) and three (42.9%) men participated. The mean age was 21.6 years (range 21–23).

Such internships are special, as there are limited places and they are concentrated in time, so the selection of students is important.

### 2.2. Study Design

The practicums were carried out through a clinical rotation at the Padre Nicolás Giner Medical Center, located in the center of the city of Requena, in the Department of Loreto, Peru for two weeks, involving health campaigns in five villages with an itinerary of 110 km by the Ucayali river that lasted a week, and programs of home care for the elderly patient and community care, developing health education interventions for two more weeks.

Before the trip, the students were given basic training on local sociocultural aspects so that the culture shock had the least possible impact. When they arrived at their destination, the students put their nursing skills into practice with the patients of the health center; they also made numerous trips to attend the population of rural areas located far from the city for different health campaigns.

The autonomous work of each student included different activities related to health promotion, triage, healthcare in acute and chronic processes, homecare, preparation of records, application of specific protocols of the health center, participation in health campaigns under supervision, and the completion of a final report in which the tasks performed and the application of the nursing intervention process were explained. The RPJ was incorporated in addition to the abovementioned activities so that the student, through annotations, reflected on their daily activities and allowed the teacher to detect and correct the difficulties of each student by analyzing the situation and directing the student in a timely manner to solve problems.

On 21 November 2019, a training session on the reflective practice journal was given by the coordinator of the practicum and the academic tutor teacher, since it was the first time it was to be used. The training session had the aim of delving into the qualities necessary for reflective thinking that are useful in the progression and acquisition of competences which, in addition, would show the perceptions of students during clinical practice.

The students answered a pre- and post-questionnaire on the training session, which showed that they all had understood the purpose of the journal.

### 2.3. Variables and Data Collection

A journal notebook was used, as it was considered the best way to preserve the privacy of the student. They filled in their RPJ as soon as they could after the end of their practicum day with a view to collecting the most significant and detailed information. The teacher accessed the journal weekly, exchanging opinions at a weekly tutorial with each student, thereby preserving privacy.

A qualitative and quantitative methodology of the experience was used taking into consideration the experience of tutors and supervisors of practicums designed by teachers who initiated the project. The journal itself was used to collect samples, questionnaires, and anonymous surveys. The diary was designed according to the scheme shown in Table 1.

Each category had one to several guiding dimensions, which were prepared considering the specific competences of the primary healthcare clinical practicum and the expected learning outcomes. The students responded to the different dimensions for 4 weeks, thus encouraging reflection on their progress [13].

On a daily basis, the students recorded their self-perception of the learning experience. For this, some notebooks were prepared where they had to select a scale of emoticons (from very sad to very happy), according to their opinion of how good or how bad their internship learning experience had been, each day during their stay. They also had a section for brief notes where they could freely express what they thought at the end of the day according to the following phases:

- 1st phase: They freely think about an aspect or category including feelings and emotions, as well as thoughts that emerged after the practices.
- 2nd phase: They deepen their knowledge and daily learning by analyzing specific situations and cases.
- 3rd phase: Discussion with the tutor on a weekly basis, where the student exposes what they have registered in the RPJ, and a critical dialogue is created to correct the aspects to improve and optimize the progression in the acquisition of knowledge.

The students tested two classmates double-blind on the last day of their stay at the duty station. They evaluated separately to preserve privacy and randomly assigned two companions per student, submitting two separate rubrics. The Likert-type rubric was made up of eight items, the lowest score being 0 (strongly disagree), then 1 (disagreeing), 2 (neutral), 3 (agree), and the maximum being 4 (strongly agree) (Table 2).

### 2.4. Data Analysis

A phenomenological qualitative analysis of the reflections written by the students in their diaries was carried out through a content analysis by reading and rereading each RPJ as a whole and selecting significant statements, which were grouped and compared according to the categories described in Table 1 to establish similarities and differences.

### 2.5. Ethical Aspects

This work was approved by the Research Ethics Committee of the Catholic University of Valencia, number UCV/2020-2021/002 and was carried out in accordance with the ethical standards established in the Declaration of Helsinki. All students signed the written informed consent for the use of their RPJ for research purposes. We followed the guidelines for research integrity stated in [14].

## 3. Results

The guiding dimensions in each category helped the students by allowing for directed reflection. Students stated that they were more aware of their clinical practice, their personal experiences and their learning progression. Reflection on an event is a deliberate and conscious activity, so in future situations similar to those experienced, it would allow them to recognize the correct way to act [4].

### 3.1. Category “Nursing Procedures”

The students agree on the adaptation of the techniques to the scarcity of means of the development environment, on the humanity necessary to be able to develop them, on the need to adapt to the local culture, and to learn as much as possible about the environment in order to apply the procedures in the best possible way.

*“It has caught my attention how they can perform procedures with everyday objects in the absence of others”, “from a plastic bottle how an air chamber is created for the administration of salbutamol. In cases of scarcity of resources, imagination is a tool to solve them” (ASJ).*

*“Today we have treated people in their homes, pure and simple nursing, we have seen that they need a lot of help. Young people are unconcerned about their elders, and it is the neighbours who are attentive to their needs. The human quality is present in those moments” (JS).*

*“In home visits, it is necessary to create a calm climate that allows fluidity and comfort to be more efficient.” “If at the moment it’s observed that there is little coordination, it’s necessary to change the way of doing things to be more productive” (ASJ).*

*“I have learned that, despite the most extreme poverty, they are happy with their families”(AMG).*

### 3.2. Category “Social Context”

When asked to reflect on the international context of practices, they agreed to recognize the clash with reality and the vulnerability of the elderly population. The Peruvian culture caught their attention, but, above all, they noticed the customs of the small towns far from the city and how they help each other. Everyone stated that the health system has enormous deficiencies, and the population consumes drugs *“as if they were candy” (ASJ)*.

*“You have to adapt to their way of life, language, lifestyle, customs, everything. Not only to integrate into society, but so that the patient is comfortable when he is cared for” (ASJ).*

*“Accustomed, possibly to a more agile health system and with much shorter response times, I believed that they would have a short wait to be transferred to a hospital” (JS).*

*“People there are abandoned by the government, because being a less accessible area, it is not taken into account and they are displaced” (AMG).*

*“The ease of accessing antibiotics without a prescription means that in a few years there will be multi-resistance to antibiotic therapy” (PE).*

*“I was struck by the lack of resources, the willingness of people to attend a talk immediately. That antibiotics are sold in the market without any type of control” (AM).*

### 3.3. Category “Motivation”

Motivation was high from the moment the students chose to carry out their internships in an international and impoverished environment. They all had in common the desire to help, to care, and to learn in another way, leaving their comfort zone. From the beginning, an adequate work environment was fostered, as well as a good relationship between the tutor and the students, with the rules and flexibility coexisting. It was important to carry out the reflective learning strategy, showing a proactive and very responsible attitude at all times.

*“Neither I nor any of my colleagues would be here if it weren’t for something moving inside us that makes us want to discover more and go a little further” (PE).*

*“I feel very motivated and excited to do nursing interventions here, see the differences and learn from them” (NS).*

*“Leaving the routine, starting to work out of the ordinary, beyond nervousness, fear and other sensations related to contact with the unknown, a new stage is usually driven by motivation” (AC).*

*“The faces of the children and mothers motivate me to continue learning, working and fighting for what I like the most, which is nursing” (JS).*

*“I attended a delivery and the mother asked my permission to put my name on her daughter, I can’t stop crying, I don’t fit the happiness I feel. Thank you life, thank you, Peru, thank you, Mayte, thank you to my parents for giving me the opportunity to live... and thank you WORLD” (JS).*

*“You want to learn absolutely everything possible.” (BG)*

### 3.4. Category “Attention to the Community”

During the first week, the students did not know how to communicate effectively with people in the community because they did not understand the language used or the meaning of the words. Despite speaking Spanish, there are differences. Sometimes understanding was difficult due to the little culture they have. As the days went by, they were able to offer talks on health promotion and risk prevention to different groups that they had to adapt to, providing them with great satisfaction. The home visits were carried out correctly, applying assessment scales and comprehensive care.

*“It has been difficult, at times, to pass scales to illiterate people since they answered yes or no without any criteria. This situation made me feel frustrated” (BG).*

*“I consider it essential to make an effort to empathize and offer confidence to obtain more reliable data when filling in rating scales” (ASJ).*

*“Thanks to the domiciliary, we were able to see what needs the patients had and we elaborated care plans adapted to them” (PE).*

*“Thanks to the theoretical and practical knowledge of the 4 years of career plus acquired knowledge, I tried to explain and put into practice the healthy lifestyle to different people. Also, those who suffered from chronic diseases and vulnerable people (the elderly, children, pregnant women, etc.)” (BG).*

### 3.5. Category “Learning”

Evidence of new aspects of learning have been left in the DRP. In general, students show a great capacity to learn independently, although it is also observed that they have supported each other in situations of doubt. The problems that have arisen have been related to lack of experience in carrying out nursing procedures with hardly any material resources, not knowing how to interview a patient, and communication with the population, due to lack of organization in health campaigns, among others. They were struck by the very rudimentary form of nursing records where the nursing taxonomy is not used. However, they realized how important it is to not waste medical supplies, although in our country we have it in abundance, now they value it much more. The skills to offer holistic care to people of different ages were acquired little by little.

*“From the first moment I have felt autonomy, from the moment I receive the patient until I say goodbye to them” (BG).*

*“I have filled out rating scales keeping an order. At first, I needed help due to my lack of experience, little by little I have been acquiring more practice. These records help us to know in what situation the population is and to be able to plan appropriate interventions to the needs” (JS).*

*“Due to the shortage of material, we had to perform a slightly modified technique” (AM).*

*“I have learned to offer recommendations, talks, to be more imaginative, more empathetic without expecting anything in return and to offer all the help that was within my reach” (NS).*

*“We have specifically planned care, taking into account the situation of people in all areas” (AC).*

### 3.6. Category “Teamwork”

The relationship between the group was very good despite the fact that the students were very different. Group work is enhanced during the degree and there was no difficulty, the effort of all to coexist and participate in all the activities was observed, and the degree of adaptation to the criticism of the teacher and classmates was extraordinarily good. All students showed skills for interpersonal relationships.

*“They began to trust us when they saw that we were asserting ourselves. With the staff of the health centre, the work and communication have been very good, we were a team, without distinctions” (PE).*

*“I have tried to make an effort to work as a team by maintaining adequate communication. I have to try harder, because the irrational fear of not doing the job correctly prevents me at times” (ASJ).*

*“Throughout the trip, I was improving communication both with colleagues and patients, their families and various social groups. Thanks to the help of my classmates, I increased my social skills” (BG).*

*“Teamwork and collaboration of all health personnel in the different activities carried out at the health post is very important. The work of each one of us is fundamental in the service to the community” (JS).*

### 3.7. Category “Suggestions for Improvement”

The students made suggestions for improvement, especially on the organization of the work to be carried out by the local health workers. The group of students suggested the convenience of increasing the hours of practicum to carry out a follow-up of the interventions carried out.

*“It would be convenient to work on women’s health issues with the general population and not only with the midwives of each community, although it is a taboo subject in that area of Peru” (ASJ).*

*“This practicum would improve only by increasing the hours, if possible, of home care, which could allow covering a greater number of elderly people, in our case, for the study of their situation in the Requena community; in addition to trying to promote health education through health campaigns in a greater number of areas, mainly in those that are furthest from civilization” (AC).*

*“Improve communication prior to mobility with the personnel responsible for the health centre, due to the complexity and ignorance and the needs of the population” (ASJ).*

*“In the tutorials that take place before the next trip with the students, I would invite some (or all) of us who have gone this year to have student-to-student referrals. I think it is beneficial” (JS).*

*“Eliminate the memory of practices and keep only the reflective diary since it consists of the daily monitoring of our practices and implies constancy and shows aspects beyond the usual ones of a memory, such as personal evolution, feelings that arise in this experience. The value of the newspaper is much higher since it allows us to fully assess this experience and not in a general way” (NS).*

The teacher was able to detect and analyze specific situations, promoting improvement changes and personalized teaching. Communication between the two was close, constant, and trustworthy. It served to help the teacher know the results of learning and competence development during the student’s stay in the Amazon and made it possible to make the appropriate adjustments. In addition, the teacher suggested a greater depth of self-criticism based on the results that each of the students was achieving. It was a positive experience because real-time guidelines and strategies were set for the care of the people.

In the self-evaluation carried out by each student in relation to knowledge and skills, the result was an average of 9.55/10, which indicates very high satisfaction. The students added numerous comments in which they expressed their limitations and insecurities, and analyzed their way of acting in specific situations with the Peruvian population.

**Figure 1:**
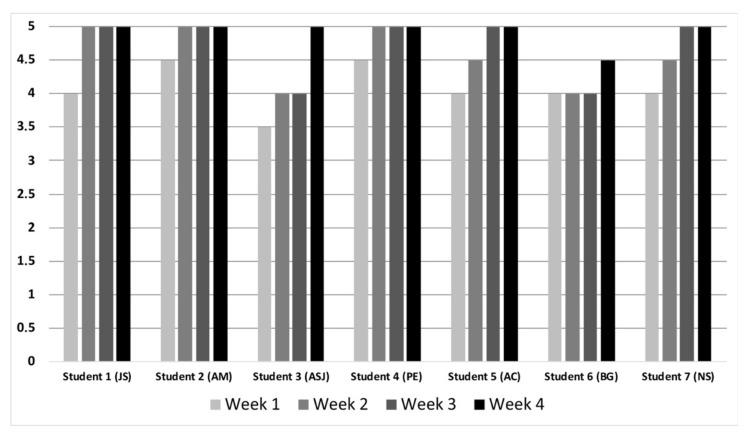
Weekly score in student self-criticism.

Every day, the teacher evaluated the student’s ability to discern and recognize their own limitations, their performance in specific situations, the difficulties they had encountered, and the reflections in their journal at the meetings held at the end of the day. As the students acquired this skill, it was observed how they were deepening their reflections and expressing how they would proceed in future situations [15]. The expressions were appropriate and very respectful. The average score determined for self-criticism was 4.57 out of 5, with 6 of the 7 students having obtained the maximum score in the previous week (Figure 1).

An anonymous satisfaction survey with the practicum and trust with the teacher at the end of the semester was made, and it indicated that they freely provided suggestions for improvement. Not performing specific nursing techniques or data collection in the community (scales or health diagnosis) was not perceived positively. The high scores were related to the work at the medical center and health education interventions in first aid and cardiopulmonary resuscitation. We observed that the students felt frustrated by the deficiencies found in the Peruvian medical center. Many days were rated with the highest score, coinciding with activities and tasks related to health campaigns and health promotion interventions. The highest scores related to practicums with the elderly, sponsorship, health campaigns, care for women in labor, and training for health promoters. Low evaluations were related to fatigue and to the observation of different ways of working to which we are accustomed. Similarly, low scores were related to being ill which made them feel guilty for not being able to provide the service that was planned. In addition, they were frustrated by having limited freedom of movement for security reasons, especially the females. We observed that some of the students felt bad the first few days when they were away from their family. With the exception of one case, all the students were very satisfied with their practicums by the end of the stay.

In the anonymous internship satisfaction survey, 100% of the students gave the highest rating to the item “I have established trust with the teacher”. The seven participating students responded to this item with the maximum rating of “totally agree”, 10/10.

The academic tutor teacher evaluated the contents collected in the journal, the reflections, and specific situations weekly, evaluating the quality of the learning experience, giving an average value of 9.46 out of 10 (minimum of 8.5 and maximum of 10).

The results of the evaluation of their practicums performance and the care offered to the population, adapting to the impoverished international context, were very high: 9.68 out of 10. Four students were evaluated with the highest grade (4/4). The remaining evaluations were 3.88/4, 3.69/4 and 3.56/4.

Given the exceptional health crisis situation caused by COVID-19, and in view of the satisfaction with the results of applying this teaching methodology, in the month of April 2020, the reflective practice journal was definitively incorporated into Module III of the portfolio educational practice of the Nursing degree.

## 4. Discussion

San Rafael [16] states that for RPJ to be useful in the evaluation of clinical practice, one part must be semi-structured, and the other have predefined structured topics. Since each student perceives situations in a particular way, it is necessary to leave blank areas so the student can write freely; as teachers we obtain much more useful information in this way. This type of journal allows the student to reflect at higher reflective levels: it is less rigid and allows more spontaneity [9,17], although an exclusively unstructured journal would lead to confusion for the student [18], and it would be necessary to program questions that provoke reflection. We believe that time passing can make one see and feel the events that occurred in a practicum day differently, so it is essential to write every day, thereby minimizing interferences.

The RPJ is not intended to merely describe events during practicums, but to reflect, deeply, critically, and sensibly, on how these occurred and how the student responded [19]. The RPJ allowed our students to consciously assess what they were writing, evaluate themselves in certain circumstances, and rethink procedures. As it is written, thought is created, and reflection is improved with the help of the tutor. Bagnato et al. [9] warns that the accompaniment and guidance by the teacher is necessary to avoid failure, and this situation was valued very positively by the seven students. Ruiz-López et al. [20] believe that RPJ can detect and solve situations of risk or conflict and favors self-learning. In our opinion, this is the case; reflecting every day and completing individual reflection with group tutoring promoted constant improvement in practical learning, making it much more attractive. There are studies of another nature [21,22] that also highlight the need for students to reflect on the clinical practicum carried out. Adequate accompaniment by the tutor is essential for progress in learning [23].

It was observed that the narrative capacity of the students increased with the passing of the days, and it was also observed that the state of illness or indisposition of the students influenced the way they perceived their practicums. Coinciding with the study by Ruiz-López and collaborators [20], the teacher became aware of situations in time to intervene, thereby guaranteeing the progression in learning and acquisition of skills, thus assuring constant improvement. In addition, it is a tool that helps the teacher evaluate the acquisition of competences and it also helps the student fill in the final report on practicums since they have been able to extract information and draw conclusions from the different sections [24]; our students were evaluated with outstanding grades and two of them were awarded honors. The RPJ allowed the students to organize and plan. It facilitated the acquisition of professional skills, improvement in the progression of learning, self-criticism, and the acquisition of values, such as hospitality, quality of care, respect, and responsibility [3,25].

The RPJ made it possible to develop the capacity for analysis and synthesis [18], for critical reasoning and management of written information, for organizing ideas, and planning tasks on successive days. Coinciding with San Rafael et al. [4], some students stated that it was an extra effort to write daily, although once the internship period was over, they valued it positively. On the other hand, there are also detractors of the RPJ, such as Domingo [26], who considers that, despite being a personal training tool, there is not enough scientific evidence to incorporate it into higher education. We believe that the RPJ should not be a tool to simply grade a student, but rather a tool to facilitate the progression in learning and the acquisition of skills.

The main limitation of this study is the small size of the sample, but this was imposed by the University’s International Practicum restrictions (the other candidates were not allowed to participate due to the risks of this experience). This limitation will be overcome with a longitudinal study made by combining data from the upcoming practicums. Another limitation, also imposed by the University, is the length of the practicum (four weeks).

This work can be useful in applying the results obtained to a greater number of students and in different subjects, specifically in the practicum, substituting the final memories for a supervised reflective diary that allows them to reflect gradually on their learning process instead of relying only on their final thoughts.

## 5. Conclusions

The results obtained show that the reflective practice journal helped students to learn. Reflexivity is a facilitating instrument of meaningful learning. The journal facilitated the acquisition of professional skills and development of values, and improved the self-criticism capacity of students. The quality of individual and group assessment improved, allowing the teacher to gain a better understanding of each student’s progress. In addition, the grades of the practicum reports were outstanding, in which affective aspects of learning have been integrated, offering a humanistic approach to our educational practice.

For us, it was a challenge to find out the student’s perception of the practical training received in a different cultural and social health context. The reflective practice journal provided a very high degree of satisfaction in this context.

Many of the current curricula for university degrees such as Nursing base their practical teaching on the adherence of the student to routine care practices in hospitals. This often leads to a lack of pedagogical–didactic strategies that include knowledge and experiences that help the student’s self-knowledge, the development of problem-solving skills, and clinical decision-making. Including the RPJ as a work tool in the study curriculum of these degrees would promote a reflective and autonomous professional exercise.

## Figures and Tables

**Table 1:** Reflective practice journal layout schematic.

Category	Dimension
Nursing procedures	Mastery of nursing procedures Acquisition of new concepts Demand for information Deficiencies found in oneself
Social context	Observation of the international context of internships
Motivation	Feel motivated Proactive attitude to learn
Attention to the community	Communication with the individual and his family. Comprehensive care for the community Promoting community health through interventions Control of health risks through detection and prevention Home care
Learning	Continuous ability to learn autonomously Problems encountered and ability to solve them Skills and knowledge Make nursing records Forecast of the necessary material prior to the practice
Teamwork	Effort to work as a team Active participation in tasks Skills for interpersonal relationships Acceptance of criticism
Suggestions for improvement	Proposals

**Table 2:** Blind peer review at global meetings.

Name of Evaluated Student: ____________________________________________________________Date: __/__/__						
Evaluating Student Name: ____________________________________________________________________________________________						
Knowledge and Skills	Strongly Agree	Agree	Neutral	Disagree	Strongly Disagree	Total Score
Provides technical and professional health care appropriate to the health needs of the people served						
Is capable of planning and providing nursing care directed to individuals, families, or groups, oriented towards health outcomes						
Is able to understand the interactive behavior of the person based on gender, group, or community, within their impoverished and multicultural social context						
Designs care plans directed at individuals, families, or groups, evaluating their impact and establishing the appropriate modifications in real time						
Promotes healthy lifestyles, self-care, supporting the maintenance of preventive and therapeutic behaviors						
Establish effective communication with patients, family, social groups, and colleagues, and promote health education fluently						
Make good use of available resources						
I have observed if there is collaboration between the different sectors of the health community and the social environment						

## Data Availability

Not applicable.
